# Trainable quantization for Speedy Spiking Neural Networks

**DOI:** 10.3389/fnins.2023.1154241

**Published:** 2023-03-03

**Authors:** Andrea Castagnetti, Alain Pegatoquet, Benoît Miramond

**Affiliations:** LEAT, Université Côte d'Azur, CNRS, Sophia Antipolis, France

**Keywords:** Spiking Neural Networks, quantization error, low latency, sparsity, direct training

## Abstract

Spiking neural networks are considered as the third generation of Artificial Neural Networks. SNNs perform computation using neurons and synapses that communicate using binary and asynchronous signals known as spikes. They have attracted significant research interest over the last years since their computing paradigm allows theoretically sparse and low-power operations. This hypothetical gain, used from the beginning of the neuromorphic research, was however limited by three main factors: the absence of an efficient learning rule competing with the one of classical deep learning, the lack of mature learning framework, and an important data processing latency finally generating energy overhead. While the first two limitations have recently been addressed in the literature, the major problem of latency is not solved yet. Indeed, information is not exchanged instantaneously between spiking neurons but gradually builds up over time as spikes are generated and propagated through the network. This paper focuses on quantization error, one of the main consequence of the SNN discrete representation of information. We argue that the quantization error is the main source of accuracy drop between ANN and SNN. In this article we propose an in-depth characterization of SNN quantization noise. We then propose a end-to-end direct learning approach based on a new trainable spiking neural model. This model allows adapting the threshold of neurons during training and implements efficient quantization strategies. This novel approach better explains the global behavior of SNNs and minimizes the quantization noise during training. The resulting SNN can be trained over a limited amount of timesteps, reducing latency, while beating state of the art accuracy and preserving high sparsity on the main datasets considered in the neuromorphic community.

## 1. Introduction

The field of neuromorphic engineering, especially Spiking neural networks (SNNs), is emerging as a new paradigm for the design of low-power and real-time information processing hardware (Abderrahmane et al., 2020). The spike information coding used by SNNs enables sparse and event-based computation through the network. The combination of these properties may lead to more energy efficient hardware implementations of neural networks, allowing state-of-the-art AI algorithms to be executed on mobile platforms with a reduced power budget (Mendez et al., 2022). However, to achieve these energy gains while simultaneously reaching the level of performance of Artificial Neural Networks (ANNs), SNNs must be able to encode analog data with high precision using very compact codes, i.e., spike trains. The encoding precision in SNN is directly related to the latency of the network. Increasing the conversion time, thus generating more spikes, lowers the quantization errors and improves performance at the cost of energy overhead. The trade-off between conversion time, i.e., latency, and performance, is an increasingly active area of research (Li et al., 2021, 2022) and the main subject of this paper. Training methods for low latency and high precision SNN can be divided in two categories: ANN-SNN conversion and direct training. ANN-SNN conversion generally lead to SNN with no accuracy degradation. However, this comes at the cost of increasing latency. At the opposite, direct training methods feature low latency, but suffered from accuracy degradation, especially on deep neural networks such as ResNet (Fang et al., 2021; Li et al., 2022).

In this paper, we propose a direct training method that can achieve both low latency and high precision thanks to Adaptive Threshold Integrate and Fire (ATIF) neurons. ATIF direct training minimizes accuracy loss compared to ANN by applying a novel trainable quantization scheme. By efficiently compressing information we can achieve high accuracy with few timesteps even for deep networks.

The main contributions of our work are listed below:

- **Quantization noise in SNN**: Spiking neurons quantize information by converting their analog inputs into sequences of spikes. We characterize the quantization error and its relationship to the different parameters of a spiking neuron.
- **Information compression through trainable quantization**: We propose a learning approach that reduces quantization error by adapting the neuron's parameters during training and using a new neural model called ATIF.
- **Low latency and sparse SNN**: We validate our approach on different image and audio classification problems, thus defining new state of the art results in terms of accuracy and latency. Moreover we show that these performance can be achieved with a significant level of sparsity. Specifically, we achieve 94.65% accuracy on CIFAR-10, and 94.31% on Google Speech Commands with less than one spike per neuron.

## 2. State of the art

In the last few years, the development of SNN has been driven by the need of matching the performance of the ANN on complex image processing tasks. Early works focused on unsupervised or semi-supervised learning algorithms based on spike timing dependent plasticity (STDP) (Diehl and Cook, 2015; Srinivasan et al., 2018). However, networks trained with STDP yield in general to considerable lower accuracy than ANN or SNN trained with backpropagation.

To take advantage of better performance provided by supervised learning, several methods have been developed to convert ANNs, trained using standard schemes like backpropagation, into SNNs for event-driven inference (Diehl et al., 2015; Rueckauer et al., 2017). The ANN-SNN conversion is based on the idea that firing rates of spiking neurons should match the activations of analog neurons. Early demonstration on complex dataset like Imagenet or CIFAR-10 showed that SNNs almost match the accuracy of ANN but at the cost of a higher latency. As an example, Sengupta et al. (2019) was able to achieve competitive results on CIFAR-10 with a latency of 2,500 timesteps.

Han et al. (2020) proposed a conversion-based training using *soft-reset* spiking neurons. The Integrate and Fire (IF) with soft-reset neuron implements a uniform quantization scheme between its analog input and its spiking output, thus leading to a reduced quantization error compared to hard-reset neurons. Moreover, to ensure that spiking neurons operate in the linear regime the authors proposed a technique to balance the firing threshold (*V*_*th*_). With the proposed model, the authors were able to achieve an accuracy of 60.30% with a latency of 32 timesteps on CIFAR-10 using a VGG-16 network. In our work, we also use the IF with soft-reset neuron model to take advantage of its uniform quantization.

Ding et al. (2021) applied a clipped ReLU during ANN training to better emulate the behavior of spiking neurons. The clipping point, which is the equivalent to the firing threshold of a spiking neuron, is trained layer-wise. After conversion, the authors obtain an accuracy of 85.40% with a latency of 32 timesteps on CIFAR-10 using a VGG-16 network.

Previous works have shown that threshold balancing clearly helps reducing the quantization error, thus decreasing the accuracy loss of SNNs. However, they fail to convert an ANN into an SNN within extremely low time steps, where quantization errors are higher. To overcome this issue, Li et al. (2021) proposed a post-training calibration pipeline that fine-tunes, layer-by-layer, the network parameters, including weights, bias and membrane potentials, therefore minimizing the local conversion error (i.e., quantization error). An accuracy of 86.57% at 4 timesteps was then obtained on CIFAR-10 using a VGG-16 network.

The authors of Li et al. (2022) went one step beyond by proposing to convert a quantized ANN to an SNN. They use a quantization-aware-training method called Learned step size quantization (LSQ) (Esser et al., 2020) to train a quantized ANN. In order to transfer the weights from the quantized ANN to the SNN, the spiking neuron model was modified to match the response curve of the quantized ReLU. However, for this method to be effective, the proposed spiking neuron must be able to generate spikes with negative polarity, which is not biologically plausible. With the previous conversion method, an accuracy of 92.64% at 4 timesteps was obtained on CIFAR-10 using a VGG-16 network. Beyond the excellent accuracy score, the authors of Li et al. (2022) have shown that there is an equivalence between quantized ANN and SNNs. Moreover, to obtain state of the art results, quantization aware training must be used to jointly optimize accuracy and quantization error.

Another method to obtain low latency SNNs is to train the spiking network directly by surrogate gradients (Neftci et al., 2019). Here, a surrogate function is used, during gradient back-propagation, to replace the binary non-linearity of spiking neurons. This allows gradient flowing thus making back-propagation possible in the spiking domain. Direct training can optimize at the same time, the network accuracy and the quantization error introduced by the spiking neurons. It can therefore be considered as a spiking-specific form of quantization aware training.

Rathi and Roy (2021) use direct learning to fine tune network parameters transferred from an ANN. They are able to achieve an accuracy of 92.70% at 5 timesteps on CIFAR-10 with VGG-16.

The authors of Fang et al. (2021) used direct learning to train ResNet without any previous conversion from ANN. They proposed SEW-ResNet, a spiking adaptation of ResNet that overcomes the vanishing/exploding gradient problem that occurs with directly trained spiking deep residual networks. They achieved an accuracy of 67.04% at 4 timesteps on Imagenet with a ResNet34. Their results show that it is possible to train deep spiking neural networks and obtain very competitive results on complex datasets.

Finally, in our work we make use of computationally efficient spiking neuron models that approximate the behavior observed in real neurons. In these models the action potentials, i.e., the spikes, are approximated using binary pulses of infinitesimally short duration. However, recent studies have shown that biological neurons can generate several spiking dynamics and they can fire spikes of different amplitudes and duration (Chakraborty et al., 2022). Moreover, in contrast to the structure of the human cortex (Panda et al., 2021), we only consider networks composed of layers of neurons with identical characteristics. The integrate-and-fire model used in this paper does take into account only a limited set of features of the biological neuron. However, it is compact and computationally efficient, thus well-suited for SNNs in the context of the machine learning tasks that we target in our work.

In this paper we propose to use direct learning to jointly optimize the network and the spiking neurons parameters. Moreover, we show that it is possible to reach or outperform state of the art results without using any non-biologically plausible artifacts, like negative polarity or non-binary spiking signals. All the results presented in this Section will be summarized in **Tables 1, 2** in Section 4.

## 3. Methods

### 3.1. Spike based information compression

Considering an n-layer fully connected or convolutional ANN, the output of the layer *l* can be described as:

(1) $$\begin{array}{ll} y^{l}=h\left(x^{l}W^{l}+b^{l}\right), & l\in \left[1,n\right] \end{array}$$

Where, *x*^*l*^, *W*^*l*^, *b*^*l*^ are the input activation, the weights and the bias of the layer *l*, respectively. Moreover, *h*(·) denotes the ReLU activation function. In SNNs the activation function is replaced with a spiking neuron, whose role is to implement the ReLU non-linearity (*max*(0, *x*)) and discretize its input signal into spikes. Here, we use the Integrate-and-Fire (IF) neuron model with soft-reset that can be described, at each timestep *t*, by the following equations:

(2) $$\begin{array}{l} H^{l}\left(t\right)=V^{l}\left(t-1\right)+i^{l}\left(t\right) \end{array}$$

(3) $$\begin{array}{l} i^{l}\left(t\right)=z^{l-1}\left(t\right)W^{l}+b^{l} \end{array}$$

(4) $$\begin{array}{l} z^{l}\left(t\right)=\Theta \left(H^{l}\left(t\right)-V_{th}\right) \end{array}$$

(5) $$\begin{array}{l} V^{l}\left(t\right)=H^{l}\left(t\right)\left(1-z^{l}\left(t\right)\right)+\left(H^{l}\left(t\right)-V_{th}\right)z^{l}\left(t\right) \end{array}$$

Where, *H*^*l*^(*t*) and *V*^*l*^(*t*) represents the membrane potential after the input integration and after the reset operation that follows the spike emission at time *t*, respectively. The spiking output at time *t* is represented by *z*^*l*^(*t*). Here, we stress the fact that *z*^*l*^(*t*) can only have binary values. The input of the spiking neuron, *i*^*l*^(*t*), can be expressed as the product of the weights with the binary signal, i.e., the spikes, generated by the preceding layer plus a constant bias. As can be seen from Equations (2) and (3), the product can be replaced with an addition of the weights with the membrane potential whenever *z*^*l*−1^(*t*) = 1. Moreover, the bias is added to the membrane potential at each timestep regardless the value of *z*^*l*−1^(*t*). Equations (4) and (5) describe the generation of a spike and the soft-reset operation, respectively. The function Θ(·) represents the Heaviside step function.

The SNN forward operation described by the previous equations, is repeated through *T* timesteps. The output of the spiking layer *l* can be decoded as follows:

(6) $$\begin{array}{l} y_{s}^{l}=\frac{1}{T}\overset{T}{\underset{t=1}{\sum }}z^{l}\left(t\right) \end{array}$$

Equation (6) defines the decoding scheme for a rate-coded network. In this scheme, the information is coded by the number of spikes generated by a spiking neuron over a fixed length of time *T*, i.e., the firing-rate. Since a spiking neuron generates spikes proportionally to its input current, the time average described in Equation (6) will converge to *y*^*l*^, when *T* → ∞. Moreover, the time integration of *z*^*l*^(*t*) which is a binary variable, leads to a quantization of the decoded output $y_{s}^{l}$. Using the preceding equations, it is possible to show that the quantization function of a IF with soft-reset spiking neuron can be expressed in the following closed form:

(7) $$\begin{array}{l} \overset{T}{\underset{t=1}{\sum }}z\left(t\right)=\min\left\{T,\frac{\sum_{t=1}^{T}\left(z^{l-1}\left(t\right)W^{l}\right)+bT}{V_{th}}\right\} \end{array}$$

**Figure 1:**
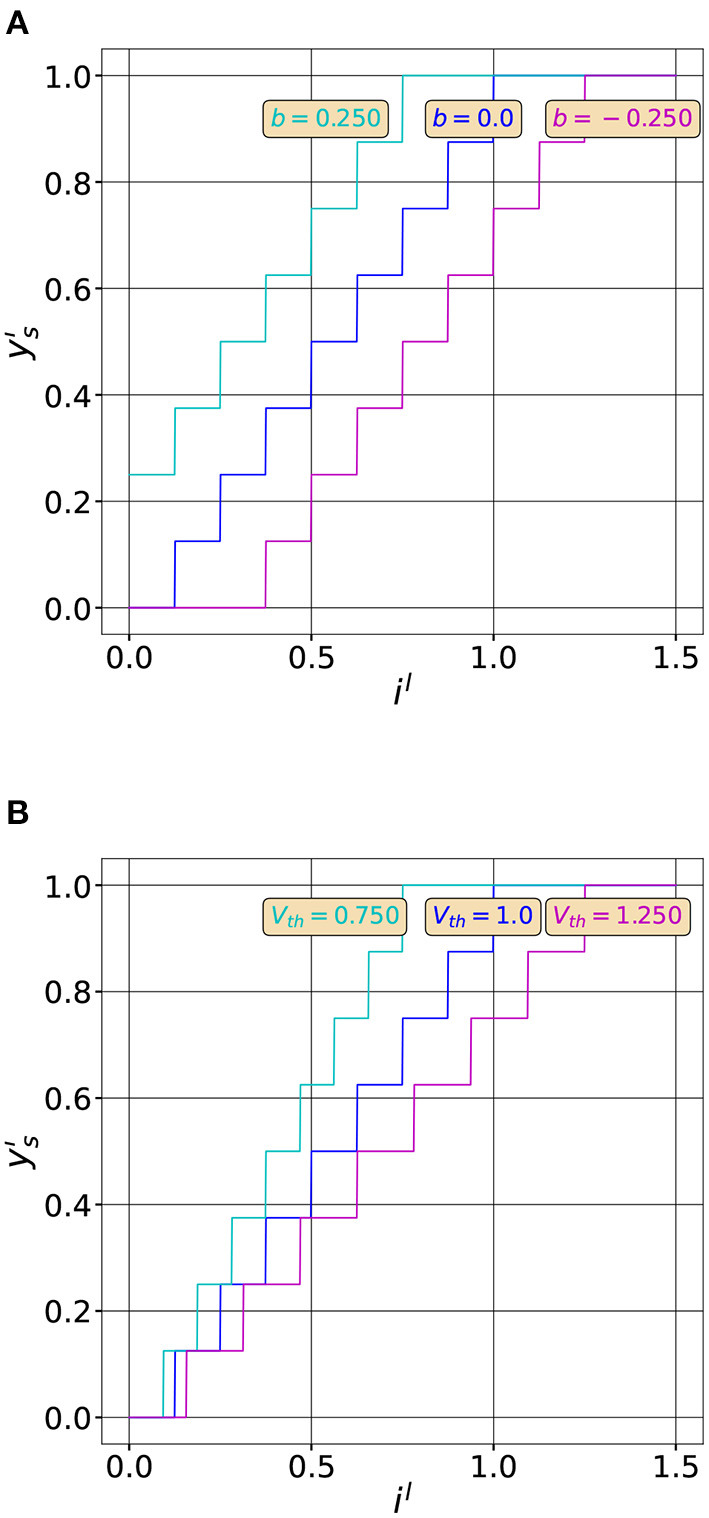
Effect of the neuron parameters on the quantization function. **(A)** Quantization functions for three different bias values (*T* = 8, *V*_*th*_ = 1.0, *b* = [0.25, 0, −0.25]). Adding a bias moves the curve in the horizontal direction. **(B)** Quantization functions for three different threshold values (*T* = 8, *V*_*th*_ = [0.75, 1.0, 1.25], *b* = 0). Modifying *V*_*th*_ changes the slope of the curve. The maximum value of the output rate (1.) is reached when $i^{l}=V_{th}$.

The numerator of Equation (7), $\sum_{t=1}^{T}\left(z^{l-1}\left(t\right)W^{l}\right)+bT$, represents the integral of the input current, noted in the following *i*^*l*^, over *T* timesteps. Figure 1 shows the effect of the different neuron parameters on the quantization function.

As we can observe, by varying the neuron parameters we can modify the quantization curve. For example, the rational behind threshold balancing is to adapt the quantization range, by modifying *V*_*th*_ to match the input distribution as shown in Figure 1B. We can also observe that the quantization function has exactly *T* + 1 uniform quantization intervals. We therefore expect a lower quantization noise by increasing the latency of the network, that is *T*. However, increasing the latency hinders the computational efficiency of SNNs. So, in order to efficiently compress information with spiking neurons and maintain, at the same time, their computational efficiency we have to reduce the quantization noise without increasing *T*. This can be achieved by optimizing the quantization function of spiking neurons to better match the input distribution of *i*^*l*^ as we will see in the next sections.

### 3.2. Quantization error analysis

Here, we characterize the quantization noise introduced by the spiking neurons. The effect of the neuron parameters and the input distribution on the quantization process are first studied. Let us consider an analog signal *x* and its quantized version $\hat{x}$. We define the quantization noise introduced during the conversion process using the Signal-to-Quantization-Noise-Ratio (SQNR) defined below:

(8) $$\begin{array}{l} SQNR\left(x\right)=10\log_{10}\left(\frac{𝔼\left[x^{2}\right]}{𝔼\left[{\left(x-\hat{x}\right)}^{2}\right]}\right) \end{array}$$

**Figure 2:**
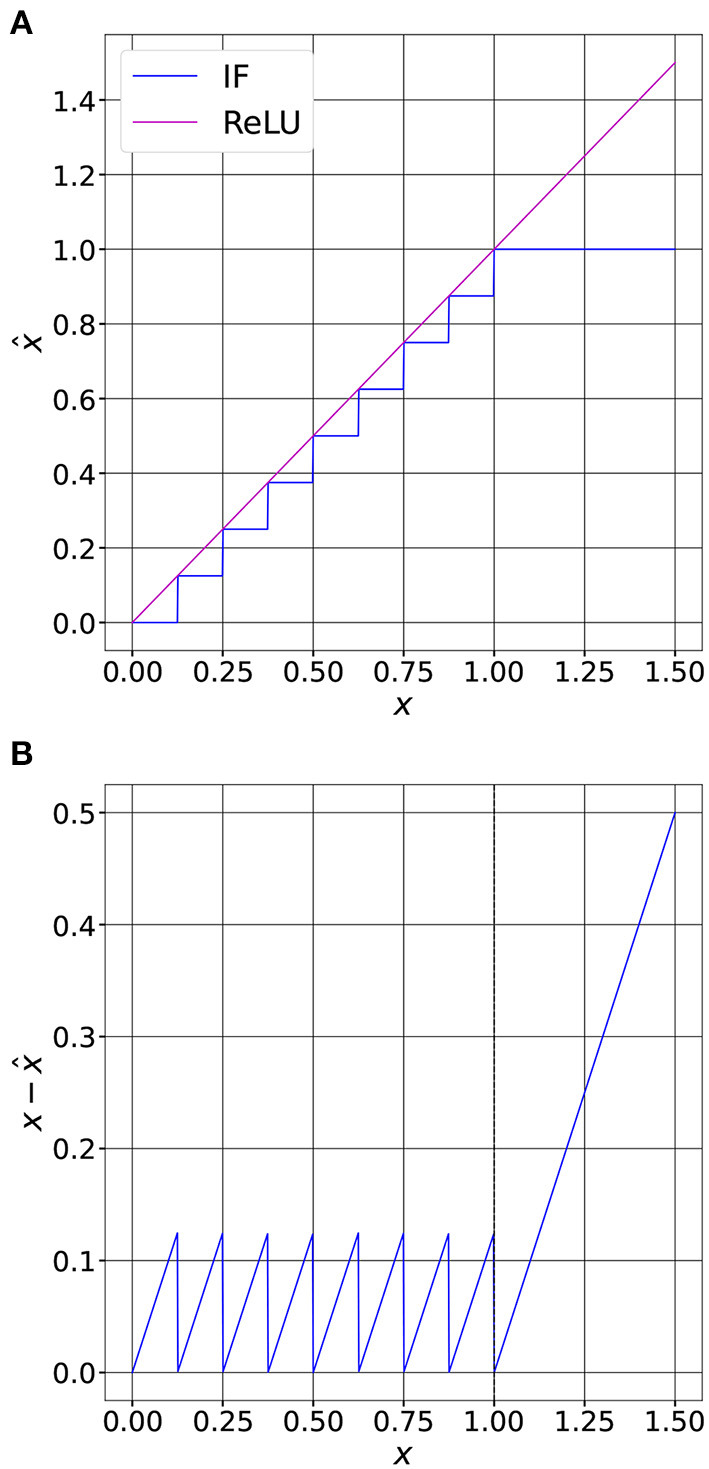
Comparison between a ReLU activation and an IF spiking neuron. **(A)** Quantization function of an IF with soft-reset (*T* = 8, *V*_*th*_ = 1.0). We can observe that the output of the neuron saturates when the input equals *V*_*th*_. **(B)** The quantization error is bounded when *x* ≤ *V*_*th*_. The bounded error is called *granular error*. When the neuron saturates, that is when *x* > *V*_*th*_, the error is unbounded and is called *overload error*.

In ANNs the ReLU activation function does not introduce any quantization noise. So, if we consider that *x* is the input of a ReLU, then *y* = *ReLU*(*x*) = *x* when *x* ≥ 0. At the opposite in SNNs, the input current of a spiking neuron is quantized as shown in Equation (7). If we define *x* = *i*^*l*^ as the input of the neuron, the decoded spiking output $y_{s}=IF\left(x\right)=\hat{x}$ is the quantized representation of its input. This process is shown in Figure 2.

From Figure 2B, we can observe that when the input of the neuron is lower than *V*_*th*_, the quantization error is bounded. At the opposite, when *x* > *V*_*th*_, the quantization error can grow without any bounds. To minimize the quantization error within a uniform quantization scheme it is thus necessary to set the saturation point, that is *V*_*th*_, to balance these two sources of error. To do so, we must know the probability density function (PDF) of the input, then optimize *V*_*th*_ to reduce the quantization noise, as we will see in the next section.

### 3.3. PDF-optimized quantization for spiking neurons

Let us consider an IF spiking neuron described by the Equations (2)–(4). In this paper, the surrogate gradient method is used to compute the derivative of the Heaviside step function during error back-propagation, that is Θ′(*x*) = σ′(*x*). Where, σ(*x*) denotes the surrogate function, i.e., an approximation of the step function. Throughout our paper, we use the sigmoid ($\sigma \left(x\right)=\frac{1}{1+e^{-x}}$) as surrogate function.

Let *V*_*th*_ be a trainable parameter of the spiking neuron, *i* and *z* its input and output, respectively. Let us call $\frac{\partial L}{\partial z}$ the upstream gradient to the neuron. The downstream gradient with respect to *V*_*th*_ can then be computed using the chain rule by multiplying the upstream gradient by the local gradient with respect to *V*_*th*_. For the sake of notation simplicity we define *q* = (*H*(*t*) − *V*_*th*_), the input of the Heaviside step function. We can then compute the downstream gradient as follows:

(9) $$\begin{array}{l} \frac{\partial L}{\partial V_{th}}=\frac{\partial L}{\partial z}\cdot \frac{\partial z}{\partial q}\cdot \frac{\partial q}{\partial V_{th}} \end{array}$$

Using Equation (4) and the derivative of the surrogate function we can compute the gradient of *z* with respect to *q* as follows:

(10) $$\begin{array}{l} \frac{\partial z}{\partial q}=\sigma^{\prime }\left(q\right) \end{array}$$

Then, from the definition of *q* we can determine:

(11) $$\begin{array}{l} \frac{\partial q}{\partial V_{th}}=-1 \end{array}$$

Finally, by replacing Equations (10) and (11) into Equation (9) we obtain the approximation of the downstream gradient:

(12) $$\begin{array}{l} \frac{\partial L}{\partial V_{th}}=-\frac{\partial L}{\partial z}\cdot \sigma^{\prime }\left(q\right) \end{array}$$

The neuron output, denoted $\hat{y}$, is computed by decoding the spiking sequence after *T* timesteps. We would like to find the value of *V*_*th*_ that minimizes the quantization error of the neuron. To do so, we analyze the optimization process for a single spiking neuron whose input current *i* follows a Gaussian distribution with zero mean, $i\sim N\left(0,\sigma^{2}\right)$. We first compare trainable and fixed *V*_*th*_ neurons with a fixed number of timesteps *T* = 4. The loss function is defined as the RMS error between the input and the output of the neuron:

(13) $$\begin{array}{l} L\left(i,\hat{y}\right)=𝔼\left[{\left(i-\hat{y}\right)}^{2}\right] \end{array}$$

As we can observe from Equation (13), by minimizing the loss function we maximize the SQNR for a given input distribution. We simulated the above optimization problem for both an IF neuron with a fixed *V*_*th*_ = 1 and an IF neuron with its threshold modified during the learning process. At each iteration, an input *i* is drawn from a Gaussian distribution with σ = 0.1. The neuron then converts the input into spikes, that are finally decoded to obtain the estimate $\hat{y}$. Once the loss is computed with Equations (13), the error is back-propagated and *V*_*th*_ (the only neuron parameter), is updated using standard gradient-based optimization (Adam optimizer, $l_{r}=10^{-3}$). In the following this model is denoted as ATIF-u.

**Figure 3:**
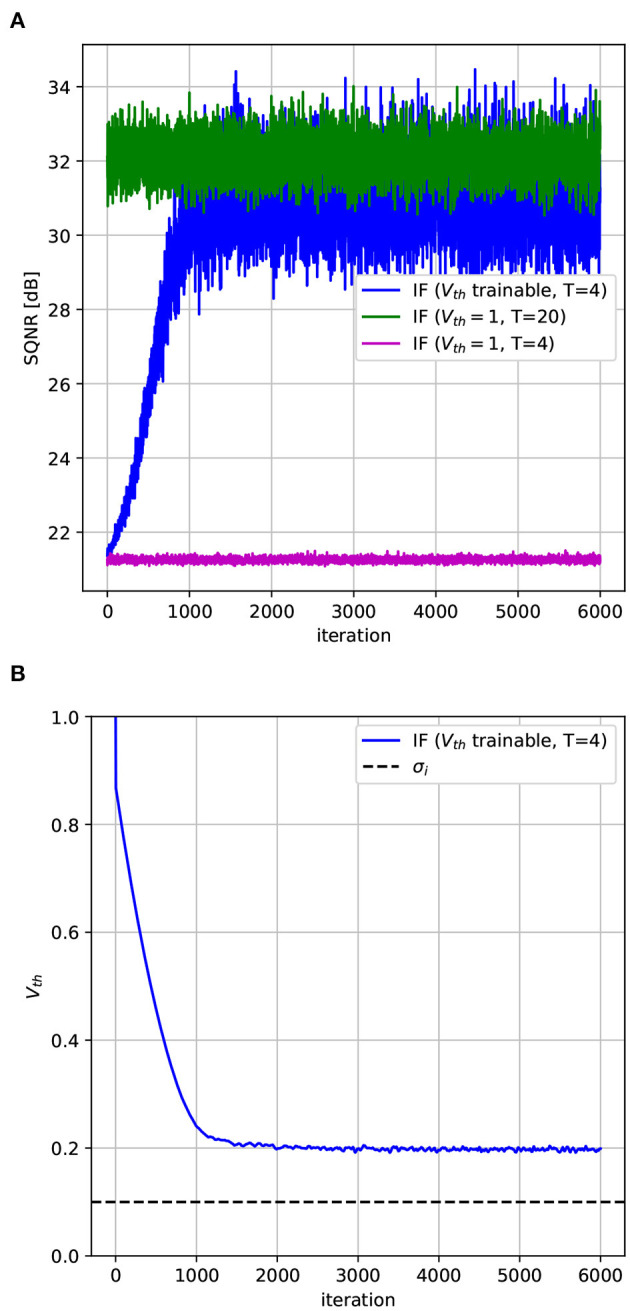
Learning the *V*_*th*_ of a spiking neuron. **(A)** SQNR of an IF with soft-reset with both trainable and fixed *V*_*th*_. Since *V*_*th*_ is modified to match the input distribution the SQNR increases during the optimization process. To obtain a similar SQNR with a fixed *V*_*th*_ we must use five times more timesteps. **(B)** *V*_*th*_ decreases during the optimization process and approaches the standard deviation of the input, σ_*i*_.

The threshold voltage is initialized to the value 1 for all neurons. The resulting SQNR is shown in Figure 3A. As it can be observed, the SQNR at the beginning of the optimization process is the same for both neurons models. While the quantization error does not vary for the model with a fixed *V*_*th*_, optimizing *V*_*th*_ can increase by more than 10 dB the SQNR compared to a fixed *V*_*th*_ model, for the same amount of timesteps (*T* = 4). From Figure 3B, we can observe that the gain in SQNR is obtained by decreasing *V*_*th*_, thus moving the quantization intervals near the region where most of the input values fall, in our case [0, 2σ_*i*_]. By optimizing the quantization function of the spiking neuron we have then been able to significantly decrease the quantization error without increasing the timesteps. As an example, to obtain the same SQNR with a fixed *V*_*th*_, we should have used five times more timesteps, that is *T* = 20, as it can be observed from Figure 3A.

We have obtained the previous results by assuming that the input of the neurons is normally distributed, which is a reasonable hypothesis for SNN and ANN in general. However, similar results and conclusions can be obtained for different type of distributions. The optimization procedure described above, that can be integrated into a Quantization-aware-training framework for SNN, is similar to some extent to what LSQ (Esser et al., 2020) did for quantized ANNs. That is, a uniform quantization scheme is tuned to match the input distribution of the neurons. We can indeed further reduce the quantization error, beyond what is possible with a uniform quantization scheme, by modifying the size of the quantization steps, thus making the quantizer non-uniform. We propose, in the next section, a modified spiking neuron model able to implement this quantization strategy.

### 3.4. PDF-optimized non-uniform quantization for spiking neurons

In the uniform quantization scheme described in the previous section, the size of each quantization step is equal to *V*_*th*_/*T*, as can be observed from Figures 1, 2. Since *V*_*th*_ is constant and does not vary during time, the number of spikes generated by the neuron only depends on the input amplitude.

**Figure 4:**
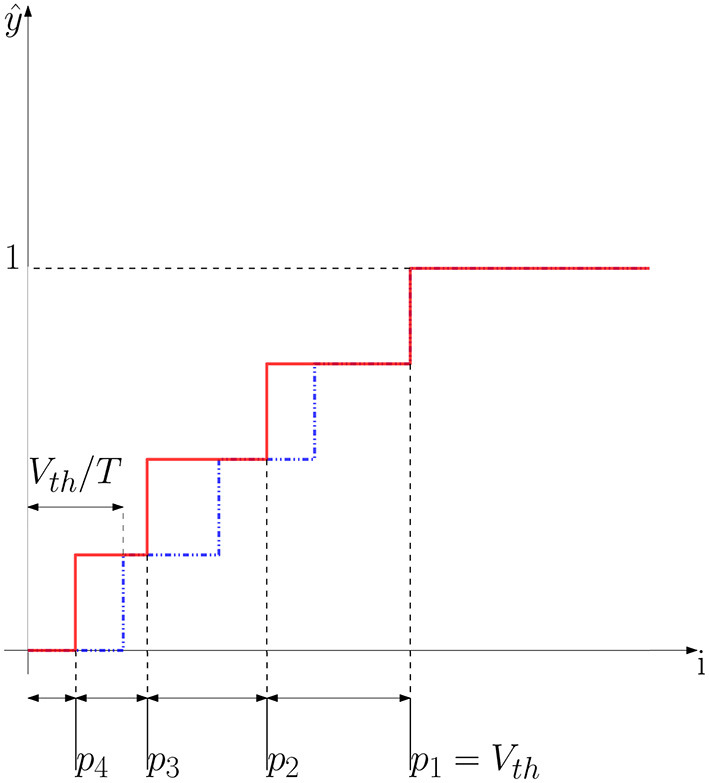
Non-uniform quantization scheme. The solid curve shows an example of a non-uniform quantizer where steps have different sizes. The dotted curve shows the uniform quantizer where all the steps have the same size *V*_*th*_/*T*. As we can observe, in the non-uniform quantization scheme the parameter *p*_1_ defines the clipping point of the quantization function, like *V*_*th*_ does in the case of an IF neuron. In this example *T* = 4 timesteps.

Figure 4 shows an example of a non-uniform quantization scheme. We can obtain this scheme by modifying the spiking neuron, precisely, we let *V*_*th*_ change during time. Let us start with an example where *T* = 4. In this case, we would like to obtain exactly four values of *V*_*th*_(*t*) = [*V*_*p*_1__, *V*_*p*_2__, *V*_*p*_3__, *V*_*p*_4__]. In the first interval, that is when *i* ≥ *p*_4_, the neuron outputs only one spike at the last timestep. Let us denote this particular output with the following notation: *z*(*t*) = [0001]. We can compute the threshold of the last timestep (*V*_*p*_4__) as follows:


(14) $$\begin{array}{l} V_{p_{4}}=p_{4}T \end{array}$$

By setting *V*_*th*_(*t* = 4) = *V*_*p*_4__ we constrain the neuron to generate a spike at the last timestep whenever an input of amplitude *p*_4_ is presented at the neuron input during *T* timesteps. We have thus set the quantization step at the value *i* = *p*_4_. Let us now consider the quantization step at value *p*_1_. When the input *i* ≥ *p*_1_, the neuron has to output the maximum rate, that is *z*(*t*) = [1111]. This condition allows us to define the threshold for the first timestep as follows:

(15) $$\begin{array}{l} V_{p_{1}}=p_{1} \end{array}$$

Following the same reasoning, when *p*_3_ ≤ *i* ≤ *p*_2_ the neuron generates a spikes in the last two timesteps, that is *z*(*t*) = [0011]. This condition can be written as follows:

(16) $$\begin{array}{l} \{\begin{array}{cc} \left(T-1\right)p_{3}\geq V_{p_{3}}, & t=T-1\\ \left(T-1\right)p_{3}-V_{p_{3}}+p_{3}=V_{p_{4}}, & t=T \end{array} \end{array}$$

The first equation in 16 describes the state of the membrane potential of the neuron at timestep *T* − 1. We set the neuron threshold at the penultimate timestep to be equal to *V*_*p*_3__ to make the neuron fire. Following a spike emission at timestep *T* − 1, the membrane potential is soft-reset, then the input *p*_3_ is accumulated at timestep *T* as shown in the second equation in 16. The equation system shown in 16 has the following solution:

(17) $$\begin{array}{l} V_{p_{3}}=T\left(p_{3}-p_{4}\right) \end{array}$$

In the same way, when *p*_2_ ≤ *i* ≤ *p*_1_, the neuron will output the following sequence *z*(*t*) = [0111] then the following three conditions are met:

(18) $$\begin{array}{l} \{\begin{array}{cc} \left(T-2\right)p_{2}\geq V_{p_{2}}, & t=T-2\\ \left(T-2\right)p_{2}-V_{p_{2}}+p_{2}\geq V_{p_{3}}, & t=T-1\\ \left(T-2\right)p_{2}-V_{p_{2}}+p_{2}-V_{p_{3}}+p_{2}=V_{p_{4}}, & t=T \end{array} \end{array}$$

Which leads us to the following solution:

(19) $$\begin{array}{l} V_{p_{2}}=\left(T-1\right)p_{2}-T\left(p_{3}-p_{4}\right) \end{array}$$

**Figure 5:**
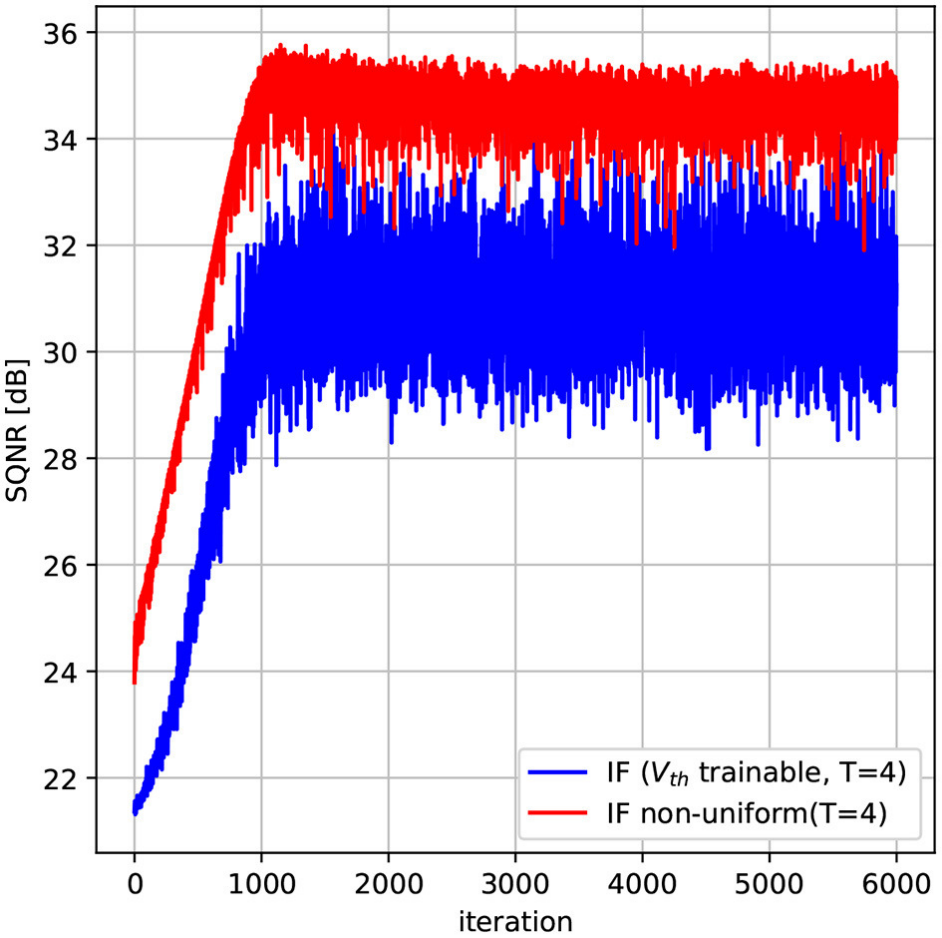
Non-uniform quantization spiking neuron. The non-uniform quantization scheme provides an increase of almost 3 dB compared to the uniform quantization scheme on a gaussian distributed input with σ = 0.1. In this example *T* = 4 timesteps.

In the following this model is denoted as ATIF-nu. We have simulated the proposed neuron model for a gaussian distributed input with σ = 0.1. The model has *T* trainable parameters, namely [*p*_1_, …, *p*_*T*_]. The thresholds are then computed using Equation (15) to Equation (19), for the case *T* = 4. We use the same optimization procedure and loss described in Section 3.3. The simulation results are shown in Figure 5 along with the uniform quantization neuron with trainable *V*_*th*_.

As it can be observed, using a non-uniform quantization scheme can increase the SQNR by almost 3dB compared to the uniform case, without increasing the number of timesteps. This gain comes from the fact that using non-uniform quantization steps, we can better approximate the input data in regions that have more probability mass. As the input of neurons in SNNs can often be modeled as gaussian sources peaked near zero, we expect that a non-uniform quantization scheme can help improving the performance of SNNs as we will see in the next section.

## 4. Experiments and results

### 4.1. Experimental setup

We trained both VGG-16 and ResNet-18 models using direct training on two different image classification problems with increasing complexity: CIFAR-10, CIFAR-100. We trained both networks with the neuron models described in Sections 3.3 and 3.4. In our setup, the threshold of neurons are trained layer-wise, so that all the spiking neurons of a given layer share the same *V*_*th*_. For each model and dataset, we also trained a formal version of the network, where spiking neuron are replaced with ReLU activation to compare SNN with a full precision ANN. Both SNN and ANN were trained using stochastic gradient descent (SGD), with a learning rate of 8 · 10^−2^. The learning rate is exponentially decayed with a factor of 0.9 each 30 epochs. Each network is trained for 900 epochs. In SNNs, the input is analog coded (Rueckauer et al., 2017), that is the spiking neurons of the first layer receive a constant input current. We use data augmentation (random resize and horizontal flip) as well as mixup with α = 1.

In addition to the image classifications problems described above, we also carried out experiments on a keyword spotting (KWS) dataset. In an automatic speech recognition system, KWS consists in detecting a relatively small set of predefined keywords. We used Google Speech Commands (GSC) (Warden, 2018) V2, a dataset of audio signals sampled at 16 kHz composed of 1-s recordings of 35 spoken keywords. Raw audio signals are pre-processed to extract Mel Frequency Cepstral Coefficients (MFCC). We used 10 MFC Coefficients, FFT of size 1024, a window size of 640 with a hop of 320, and a padding of 320 on both sides. The pre-processing generates a 48 × 10 coefficients matrix that is subsequently processed by the neural networks. We trained a Resnet-18 network using the same training configuration used for CIFAR-10/100 except for the learning rate and the number of epochs. The learning rate is initialized at 10^−3^ and exponentially decayed with a factor of 0.1 each 20 epochs. Finally, each network is trained for 80 epochs. We used PyTorch and the SpikingJelly (Fang et al., 2020) framework for simulating SNNs. In the following sections, we report the accuracy as well as the latency and the sparsity of the SNN. We measured the sparsity of our networks, that we call θ, by counting the average number of spikes generated by the spiking neurons during the inference. The sparsity of a tensor of size (*m, n*) is computed as follows:

(20) $$\begin{array}{l} \theta =\frac{\overset{T}{\underset{t=1}{\sum }}\overset{n}{\underset{i=0}{\sum }}\overset{m}{\underset{j=0}{\sum }}z_{i,j}\left(t\right)}{n\times m\times T} \end{array}$$

To report a single global sparsity, we average the sparsity of each tensor in the SNNs. Moreover, the sparsity is averaged over the entire test set that is composed of 10K images in the case of CIFAR-10. The θ parameters measure the average activity of spiking neurons. A low value means low activity and therefore a potential increase in energy efficiency of the network when deployed on a neuromorphic dedicated circuit (Lemaire et al., 2022).

### 4.2. Experimental results on CIFAR-10/100

Benchmark results on CIFAR-10/100 datasets are shown in Table 1. Our models perform consistently better than recent state of the art ANN-SNN conversion methods on both datasets. As an example, we improve the top-1 accuracy of ResNet-18 on CIFAR-10 by 1.5% with respect to QFFS (Li et al., 2022). Experimental results also show that our quantization approaches outperform previous methods regardless of the network architecture. As an example, we improve the top-1 accuracy on CIFAR-10 for both ResNet-18 and VGG-16 architectures. The non-uniform quantization scheme provides the best accuracy scores in 3 out of four configurations, while the uniform scheme provides a slightly better accuracy score on ResNet-18 and CIFAR-100. It is worth noting that, these improvements are obtained using only 4 timesteps. So, we are able to improve the classification accuracy without degrading the SNN latency compared to current state of the art methods. Besides the latency, the sparsity parameter also has a strong impact on the SNN efficiency. The amount of operations executed during SNN inference is indeed related to the average firing rate of the neurons (Lemaire et al., 2022). The sparsity parameter, θ, shows that our networks are able to classify images using on average less than one spike per neuron. As an example, for VGG-16 with a uniform quantization scheme, each neuron generates on average 0.111 × 4 = 0.44 spikes. It can also be observed that the non-uniform quantization scheme generates more spikes on average than the uniform quantizer. Since more quantization steps are allocated on the region of the input where data appears more frequently, i.e., where the PDF has higher probability mass, the generation of spikes increases accordingly.

**Table 1:** Benchmark results on CIFAR-10/100 datasets.

**Method**	**Architecture**	**ACC (ANN)**	**ACC (SNN)**	**Latency**	**θ**
**CIFAR-10**					
RMP (Han et al., 2020)^⋆^	VGG-16	93.63	60.3	32	-
	ResNet-20	91.47	91.36	2048	-
ACP (Li et al., 2021)^⋆^	VGG-16	95.6	86.57	4	-
	ResNet-20	96.72	84.70	4	-
QFFS (Li et al., 2022)^⋆^	VGG-16	92.44 (2/3 bits)	92.64	4	-
	ResNet-18	93.12 (2/3 bits)	93.14	4	-
**ATIF-u** ^†^	VGG-16	95.6	92.51	4	0.111
	ResNet-18	95.96	93.84	4	0.113
**ATIF-nu** ^†^	VGG-16	95.6	**93.13**	**4**	0.129
	ResNet-18	95.96	**94.65**	**4**	0.148
**CIFAR-100**					
RMP* (Han et al., 2020)^⋆^	VGG-16	71.22	63.76	128	-
	ResNet-20	68.72	67.82	2048	-
ACP* (Li et al., 2021)^⋆^	VGG-16	77.93	55.60	4	-
	ResNet-20	81.51	54.96	4	-
**ATIF-u** ^†^	VGG-16	74.47	66.54	4	0.159
	ResNet-18	74.35	**71.42**	**4**	0.192
**ATIF-nu** ^†^	VGG-16	74.47	**66.92**	**4**	0.167
	ResNet-18	74.35	70.83	4	0.191

Benchmark results on CIFAR-10/100 datasets. Best accuracy results for both ResNet and VGG-16 networks are highlighted in bold. Symbol

⋆ denotes ANN-to-SNN conversion methods while

† denotes surrogate gradient learning, i.e., direct training methods.

### 4.3. Experimental results on Google Speech Commands

The experimental results on the Google Speech Commands dataset are shown in Table 2. We compared both our quantization schemes, ATIF-u and ATIF-nu, with two recently published SNNs. The first network, called Recurrent (Cramer et al., 2022), is a single layer recurrent-SNN composed of 128 Leaky-Integrate-and-Fire (LIF) neurons and trained using surrogate gradient and BPTT. E2E is based on a ResNet-8 architecture with IF spiking neurons and is trained using ANN-SNN conversion. Notably, neither networks use threshold balancing to mitigate the quantization error introduced by the spiking neurons. As we can observe from Table 2, Recurrent SNN proposed in Cramer et al. (2022), which features a very simple architecture, is not able to match current state of the art results on this particular task even with a latency of 200 timesteps. At the opposite E2E reaches an accuracy score of 92.9% using 32 timesteps. This accuracy score, which is closer to the state of the art, is nevertheless obtained at the cost of a high latency. At the opposite, our models can consistently outperform recent state of the art SNNs both in terms of accuracy and latency. As an example, we obtain an accuracy of 94.31%, which is only 0.15% below the accuracy of the full-precision ANN using only 4 timesteps. These results confirm the importance of adopting a quantization-aware-training strategy, i.e., direct learning, but also to jointly optimize the spiking neurons parameters, to reduce quantization noise, as our ATIF models do.

**Table 2:** Benchmark results on Google Speech Commands V2 dataset (35 classes).

**Method**	**Architecture**	**ACC (ANN)**	**ACC (SNN)**	**Latency**	**θ**
Recurrent (Cramer et al., 2022)^^†^^	RSNN	-	50.9	200	-
E2E (Yang et al., 2022)^⋆^	ResNet-8	-	92.9	32	-
**ATIF-u** ^^†^^	ResNet-18	94.46	**94.31**	**4**	0.12
**ATIF-nu** ^^†^^	ResNet-18	94.46	94.29	4	0.12

Best accuracy results are highlighted in bold. Symbol

⋆ denotes ANN-to-SNN conversion methods while

† denotes surrogate gradient learning, i.e., direct training methods.

### 4.4. Ablation studies

We decompose the effects of our methods using an ablation study on the CIFAR-10 dataset. Three ResNet-18 networks are trained with different spiking neuron models. The baseline corresponds to a network with IF soft-reset spiking neurons, where all neurons share a fixed *V*_*th*_ = 1. We compare this network with both uniform and non-uniform quantization schemes described in Sections 3.3 and 3.4, respectively. In those schemes *V*_*th*_ is a trainable parameter: it is trained layer-wise for each network. All three networks are trained using surrogate gradient learning and use the same training setup described in Section 4.1.

As shown on Table 3, a significant accuracy improvement can be obtained by optimizing the *V*_*th*_ of spiking neurons during training. As an example, comparing the uniform and fixed threshold quantization scheme with the trainable one we can observe that the accuracy increases by 4.16%. We can also observe, that we achieve an accuracy improvement without increasing neither the latency nor the number of the generated spikes. By using a trainable *V*_*th*_ scheme, the sparsity can be further reduced, so that we can improve at the same time the performance and the computational efficiency of the network. Finally, using a nonuniform quantization scheme provides a further improvement of 0.81% on the accuracy. However using this scheme, neurons generate slightly more spikes than the uniform quantizer with trainable *V*_*th*_.

**Table 3:** The impact of *V*_*th*_ on the accuracy and the sparsity of the SNN.

**Quantizer**	***V*_*th*_**	**ACC (SNN)**	**Latency**	**θ**	**Spikes/neuron**
Uniform	Fixed	89.68	4	0.17	0.68
ATIF-u	Trainable	93.84	4	0.113	0.452
ATIF-nu	Trainable	94.65	4	0.148	0.592

Results are given for a ResNet-18 network on the CIFAR-10 dataset.

## 5. Discussion and further improvements

In this paper, we have approached the problem of SNNs latency using tools and metrics available within the data compression and information theory domains. We have shown that the source of accuracy degradation between ANN and SNN resides in the quantization error caused by the discretization of the information exchanged by neurons. By leveraging the techniques used in data compression, i.e., scalar quantization, we show that we can improve the performance of spiking neurons. Moreover, by improving the signal-to-quantization-noise ratio of the neurons we show that we can significantly boost the accuracy of the SNNs overall. Finally, our results are obtained without compromising the efficiency of the resulting SNNs. We are able to approach the performance of full precision ANNs (1.31% difference in the case of ResNet-18 on CIFAR-10 and 0.15% difference on GSC) using only 4 timesteps and 0.5 spikes/neuron on average.

The SNNs presented in this paper are rate-coded networks. Rate coding have long been considered as an inefficient coding scheme, mainly because of the huge number of required timesteps on early works (Sengupta et al., 2019) to approach ANNs accuracies. However, this inefficiency is not intrinsically related to the rate coding mechanism but rather to the quantization scheme used to encode information. Moreover, the methodology that we have used in our analysis can also be applied to other types of coding mechanisms, such as time-coded networks.

While most of the works in the literature still use conversion techniques, our SNNs were trained using a direct learning scheme. With direct learning, time is taken into account during the training process. We can therefore optimize the dynamic behavior of the network. This has some advantages over conversion schemes. For example, Li et al. (2022) identify a source of quantization error, called occasional noise, produced by the oscillation of the input current of the neurons. They reduce the impact of this noise source by introducing a mechanism to generate spikes with negative polarity. This phenomenon is not relevant when the network is trained in the spike domain. Quantization errors caused by the dynamic behavior of the network are taken into account and minimized during the training process. Therefore, we do not need to introduce non-biological plausible mechanisms in our networks.

One of the main drawbacks of the proposed method is the complexity and the memory budget required by the direct learning with surrogate gradient during training. Since each forward and backward pass must be repeated *T* times to compute the gradients, the training procedure is slower compared to the ANN-SNN conversion where only the ANN is trained, then the weights and biases are transferred to the SNN. Moreover, since direct learning uses back propagation though time (BPTT) it is prone to the vanishing and the exploding gradient problems. If the latter could be mitigated using, for example, gradient clipping the former is more difficult to deal with. With vanishing gradients the learning process of the SNNs slow down. Therefore, to avoid underfit, SNNs training requires a relative large number of epochs and generally a higher learning rate compared to ANNs.

The time dimension is a fundamental part of the SNNs paradigm. SNNs are therefore well-suited to process spatio-temporal information. Therefore, another interesting future direction could be the extension of our work for the case of SNNs processing time varying signals. Our analysis of quantization must be revisited since the input current of the neuron cannot be considered constant anymore. Finally, we only considered SNNs were weights, biases and neuronal parameters are coded using full-precision representations, e.g., floating point. To obtain full benefits from the low-complexity computational model of the SNNs on neuromorphic hardware the memory footprint of the model must also be considered. In this case it could be possible to integrate a quantization-aware-training procedure, e.g., LSQ, during SNN training to quantize the network parameters using low-precision representations.

## Data availability statement

Publicly available datasets were analyzed in this study. This data can be found here: https://www.cs.toronto.edu/kriz/cifar.html, https://www.tensorflow.org/datasets/catalog/speech_commands.

## Author contributions

AC developed the methods, under the supervision of AP and BM. All authors contributed to the article and approved the submitted version.
